# Data-Driven, Visual Framework for the Characterization of Aphasias Across Stroke, Post-resective, and Neurodegenerative Disorders Over Time

**DOI:** 10.3389/fneur.2020.616764

**Published:** 2020-12-29

**Authors:** Joline M. Fan, Maria Luisa Gorno-Tempini, Nina F. Dronkers, Bruce L. Miller, Mitchel S. Berger, Edward F. Chang

**Affiliations:** ^1^Department of Neurology, University of California, San Francisco, San Francisco, CA, United States; ^2^Department of Psychology, University of California, Berkeley, Berkeley, CA, United States; ^3^Department of Neurology, University of California, Davis, Davis, CA, United States; ^4^Department of Neurological Surgery, University of California, San Francisco, San Francisco, CA, United States

**Keywords:** trajectories, principal component analyses, stroke, primary progressive aphasia, aphasia

## Abstract

Aphasia classifications and specialized language batteries differ across the fields of neurodegenerative disorders and lesional brain injuries, resulting in difficult comparisons of language deficits across etiologies. In this study, we present a simplified framework, in which a widely-used aphasia battery captures clinical clusters across disease etiologies and provides a quantitative and visual method to characterize and track patients over time. The framework is used to evaluate populations representing three disease etiologies: stroke, primary progressive aphasia (PPA), and post-operative aphasia. A total of 330 patients across three populations with cerebral injury leading to aphasia were investigated, including 76 patients with stroke, 107 patients meeting criteria for PPA, and 147 patients following left hemispheric resective surgery. Western Aphasia Battery (WAB) measures (Information Content, Fluency, answering Yes/No questions, Auditory Word Recognition, Sequential Commands, and Repetition) were collected across the three populations and analyzed to develop a multi-dimensional aphasia model using dimensionality reduction techniques. Two orthogonal dimensions were found to explain 87% of the variance across aphasia phenotypes and three disease etiologies. The first dimension reflects shared weighting across aphasia subscores and correlated with aphasia severity. The second dimension incorporates fluency and comprehension, thereby separating Wernicke's from Broca's aphasia, and the non-fluent/agrammatic from semantic PPA variants. Clusters representing clinical classifications, including late PPA presentations, were preserved within the two-dimensional space. Early PPA presentations were not classifiable, as specialized batteries are needed for phenotyping. Longitudinal data was further used to visualize the trajectory of aphasias during recovery or disease progression, including the rapid recovery of post-operative aphasic patients. This method has implications for the conceptualization of aphasia as a spectrum disorder across different disease etiology and may serve as a framework to track the trajectories of aphasia progression and recovery.

## Introduction

Aphasias are acquired language disorders that are caused by injury to language areas of the brain. Common etiologies include stroke, tumors, traumatic brain injuries, or neurodegeneration. While each of these conditions can lead to dysfunction of language processing, an outstanding question is how the behavioral symptoms of these conditions relate to one another. Here, we provide a simplified, two-dimensional framework, by which aphasia subtypes can be compared across etiologies and tracked over time.

The understanding of language networks has predominantly been developed from stroke and neurodegenerative cases, which have largely been studied independently (1–3). While stroke leads to a pattern of injury that abides to a vascular distribution, neurodegenerative processes often lead to more diffuse injuries that cross vascular territories and affect regions rarely isolated in stroke (4–6). Lesional studies are often characterized using classical nomenclature, i.e., Broca's, Wernicke's, conduction, transcortical motor and sensory, anomic, and global aphasia. On the other hand, given the unique language profile and selective vulnerability of specific language networks in neurodegenerative disorders, a separate clinical classification scheme has been developed for PPA (7–9). The PPA variants currently recognized include non-fluent/agrammatic (nfvPPA), semantic (svPPA), and logopenic variants (lvPPA) (8, 10–12). More recently, transient aphasias have been found to occur in over 70% of patients undergoing left hemisphere peri-sylvian resection (13–15), offering yet another approach for studying language dysfunction.

A method to unify behavioral descriptions is an important step to help with comparisons across disease etiologies, which provide opportunity to extend coverage of complex language networks. An example was the routine exclusion of the anterior temporal lobe from the stroke-based aphasia literature before the study of svPPA (6, 16). Cross-etiology studies have been limited, as patients of each disorder are typically evaluated in separate contexts and with disease-specific assessments, in addition to their disease specific classifications. In addition, simplified methods to characterize aphasia over time may supplement ongoing efforts to understand disease trajectories (17), such as with stroke recovery (18, 19) or neurodegenerative progression (20).

In this study, we had the opportunity to analyze data acquired from individuals suffering from three disease etiologies: stroke, PPA, and transient post-resective aphasias by employing a commonly-used aphasia assessment tool, the Western Aphasia Battery (WAB) (21). First, using data-driven techniques, we developed a model based on cross-sectional data from multiple disease etiologies, as to form a common behavioral platform. Second, using this framework, we demonstrate that disease phenotypes can be visually tracked over time. Recent efforts (22) have demonstrated the feasibility of comparing across patient cohorts using dimensionality reduction techniques; we propose an alternative two-dimensional platform and demonstrate the feasibility of tracking disease attributes over time. Ultimately, this method, combined with more detailed language evaluations shared across cohorts may allow for the interdisciplinary study of complex language networks, by focusing on their similarities rather than their differences. Furthermore, it can enable the comparison and visualization of phenotypes over time in an intuitive and rapid manner for clinical utility.

## Materials and Methods

### Patient Cohort

Three patient cohorts were retrospectively examined in this study (total *n* = 330). The first cohort was comprised of 147 patients undergoing left hemisphere resective surgery between September 2010 and October 2013 at the University of California, San Francisco (UCSF) Medical Center. Inclusion criteria included patients with (1) left hemisphere resective surgery; (2) left hemisphere dominance for language as confirmed by the Wada test or pre-surgical language deficits; (3) English proficiency; and (4) administration of the WAB 2–3 days post-surgery. Of the 147 patients tested 2–3 days post-operatively, 85 patients were also assessed 1 month post-operatively and included in the disease progression analysis. Patients underwent neurosurgical resection for a range of indications, including malignancy, epileptogenic foci, and vascular malformations.

The second cohort comprised of 107 English-proficient patients with known neurodegenerative disorders undergoing language evaluation at UCSF with the WAB between July 2001 and October 2014. Of 107 patients, 75 patients had clinically classified primary progressive aphasia and were classified into non-fluent/agrammatic, semantic, and logopenic variants based on the international consensus recommendations (8). Of the 75 patients with PPAs, 50 patients were tested at a later time and included in the disease progression analysis.

The third cohort consisted of 76 English-speaking patients with single left hemisphere strokes leading to language dysfunction. These patients underwent language assessments between June 1988 and May 2009 at the VA Northern California Health Care System (VANCHCS). Of the 76 patients, 70 were classifiable by the WAB criteria. All patients were tested on multiple occasions and included in the disease progression analysis. These data were provided via a Cooperative Research and Development Agreement between the VANCHCS and UCSF.

All patients whose de-identified data were used in this study gave written consent under the original protocols from which these data were derived, and all studies had been approved by local Human Research Protection Programs.

### Aphasia Battery Examination

In each cohort, the WAB was administered by a speech language pathologist or a trained research assistant. The WAB (21) is a comprehensive and validated aphasia battery that assesses five language domains: fluency, information content, auditory comprehension, repetition, and naming. Fluency is rated by the clinician on a subjective scale based on the flow of the patient's spontaneous speech output and the degree of grammatical structure produced. Information content assesses the functional value of the patient's speech, including accuracy of responses to basic questions. Auditory comprehension is assessed by responses to yes/no questions and the comprehension of single words and sequential commands containing various degrees of grammatical complexity. The repetition task incorporates the repetition of words, phrases, and sentences. Finally, the naming task evaluates object naming, semantic fluency, sentence completion, and responsive speech tasks.

The primary metrics used in this study were the six individual WAB subtest scores that were shared across all three patient cohorts, thus allowing for the direct comparison of data across the three etiologies. These included: content, fluency, yes/no comprehension, auditory word recognition, sequential commands, and repetition. Clinical classifications for the stroke and resection cohort data were made based on the WAB classification criteria (21). For the classification of PPA, the most currently-accepted clinical criteria were used (8). In this group, the full WAB battery was not administered, as naming abilities were assessed with alternative measures.

### Statistical Methods

Dimensionality reduction techniques were utilized to visualize multi-dimensional data along dimensions that capture the highest variability in the data. In this study, principal component analysis (PCA) was used to construct dimensions from linear combinations of features across all data sets. Core datasets across all three cohorts were combined to arrive at a common basis, or a set of new dimensions, such that visual comparisons could be made across patient cohorts using the same basis. Within the core dataset, each patient was represented once, as determined by the expected evolution of the cohort. For instance, for the stroke and post-resective cohorts, each patient was represented by their initial data point; for the PPA cohort, each patient was represented by the latest data point. The initial data point for all post-resective patients was represented by testing on post-operative day 2–3; the averaged last clinic data point for the PPA cohort was 74.5 months (SD = 26.1, median 74).

Raw patient data was then normalized to the mean and standard deviations of the combined data set and then projected onto the common basis. The new dimensions were ordered by the explanatory power for variance in the data; i.e., Dimension 1 explaining the most variance, and Dimension 6 explaining the least variance. *T*-tests were performed to demonstrate separability of clinical cohorts across individual dimensions or summary metrics. Longitudinal data was projected onto the common bases constructed by the original data, which includes patients both with and without longitudinal data.

Given that the determination of principal components (PCs) can be strongly influenced by variation in sample population (23), a weighted PCA (24, 25) was also performed to account for non-uniformities in patient sampling and to ensure equal representation of clinically distinct aphasia types, regardless of disease etiology. The methods and findings are detailed in the Supplementary Table 1 and Supplementary Figure 1. Given the similarity of results between the two techniques, including the relative relationships between aphasia clusters and the variability explained in the data, the findings from the non-weighted PCA is henceforth used for demonstration.

## Results

### Cross-Sectional Study: Characterization of Aphasia Phenotypes via a Multi-Dimensional Framework Across Multiple Disease Etiologies

In order to develop a framework generalizable to all three disease etiologies, data from all three cohorts were included to derive a common basis. The clinical assessments performed across all three patient cohorts are presented in Table 1. For the stroke and post-resective patient cohorts, the WAB assessment was performed in full to determine the aphasia classification and aphasia quotient (AQ). The AQ is a weighted average of WAB subscores and represents aphasia severity (26). For the PPA cohort, the WAB assessment was not performed in full, and the AQ was thus not calculated. PCA was performed on the aggregated data in Table 1 to arrive at a common basis representing the most salient aphasia features across three disease etiologies. The contributions of the WAB subscores to each dimension are illustrated in Table 2, in which the dimensions are ordered by the degree of variability captured within the data. Dimension 1 explained 75.0% of the variability in the data, while dimension 2 accounted for 12.0%. Dimensions 3 and 4 captured 4.0 and 3.6%, respectively. For comparison, weighted PCA was also performed on aggregated data; the loadings are displayed in Supplementary Figure 1.

**Table 1:** WAB assessments for neurodegenerative, stroke and post-resective patient cohorts depicting the time points of highest pathology.

		**#Pts**	**Content**	**Fluency**	**Y/N**	**Word rec**	**Seq commands**	**Repetition**	**AQ^a^**
Neurodegeneration^b^	PNFA	25	7.1 ± 3.0	5.4 ± 2.9	56.5 ± 5.0	57.2 ± 4.8	66.4 ± 14.8	65.6 ± 30.4	
	Logopenic	17	6.9 ± 2.3	6.5 ± 2.4	49.6 ± 15.9	50.1 ± 14.6	52.1 ± 25.1	59.1 ± 22.8	
	Semantic	33	6.9 ± 2.7	8.4 ± 0.8	45.0 ± 15.8	42.4 ± 14.3	52.4 ± 27.1	81.9 ± 16.2	
	Unclassifiable	32	8.4 ± 2.4	8.4 ± 2.0	56.1 ± 7.9	54.2 ± 12.5	67.2 ± 20.1	81.4 ± 21.2	
	Total	107	7.3 ± 2.6	7.4 ± 2.4	51.9 ± 12.9	51.3 ± 12.7	58.7 ± 22.3	73.5 ± 23.3	
Stroke	WNL	10	9.9 ± 0.3	9.6 ± 0.5	59.7 ± 0.9	59.8 ± 0.6	79.6 ± 1.2	98.8 ± 2.4	97.4 ± 2.5
	Anomic	20	8.6 ± 0.9	8.0 ± 1.7	58.8 ± 2.4	56.6 ± 6.4	66.4 ± 15.6	91.5 ± 7.2	85.1 ± 7.4
	Conduction	6	8.2 ± 0.9	7.8 ± 1.8	55.5 ± 2.3	57.0 ± 2.8	64.2 ± 14.5	52.8 ± 13.5	73.4 ± 12.4
	Transcortical sensory	2	7.5 ± 1.5	6.5 ± 1.5	54.0 ± 0.0	41.0 ± 1.0	26.0 ± 1.0	89.0 ± 8.0	71.9 ± 7.4
	Wernickes	15	4.3 ± 2.6	7.5 ± 0.6	37.2 ± 14.6	29.3 ± 15.0	19.0 ± 17.8	27.6 ± 24.3	42.2 ± 18.0
	Brocas	14	4.0 ± 2.6	2.3 ± 1.4	51.2 ± 8.8	42.6 ± 12.1	48.8 ± 15.8	27.0 ± 23.3	38.7 ± 19.7
	Global	3	0.0 ± 0.0	0.3 ± 0.5	29.0 ± 20.5	14.0 ± 7.8	2.0 ± 2.8	0.0 ± 0.0	5.6 ± 3.1
	Unclassifiable	6	9.2 ± 0.4	7.3 ± 2.1	58.0 ± 2.2	59.0 ± 1.5	71.3 ± 7.4	80.7 ± 19.1	86.1 ± 9.2
	Total	76	6.7 ± 3.2	6.6 ± 3.0	51.6 ± 12.8	47.2 ± 16.1	52.1 ± 26.9	60.4 ± 36.7	65.4 ± 28.7
Post-resective	WNL	49	10.0 ± 0.1	9.5 ± 0.5	59.4 ± 1.4	59.9 ± 0.4	78.4 ± 2.7	96.2 ± 4.0	97.2 ± 1.9
	Anomic	46	9.0 ± 1.3	8.2 ± 1.2	55.9 ± 4.6	56.9 ± 3.4	67.8 ± 9.1	85.8 ± 8.3	84.9 ± 7.6
	Conduction	13	8.2 ± 1.8	7.7 ± 1.5	53.5 ± 2.8	55.2 ± 3.3	59.9 ± 9.4	57.7 ± 11.7	74.5 ± 9.8
	Transcortical motor	4	4.8 ± 1.1	2.5 ± 0.5	56.2 ± 2.5	42.8 ± 15.9	47.5 ± 15.6	80.0 ± 26.7	57.4 ± 9.4
	Wernickes	10	5.5 ± 2.3	6.5 ± 1.3	37.2 ± 9.0	33.1 ± 13.2	30.2 ± 15.8	30.6 ± 23.3	44.3 ± 13.8
	Brocas	15	4.1 ± 2.6	2.1 ± 1.7	50.8 ± 8.9	49.2 ± 9.9	46.2 ± 18.2	32.5 ± 32.6	40.0 ± 22.0
	Global	10	0.7 ± 1.0	0.9 ± 1.2	13.8 ± 18.2	3.3 ± 4.9	0.8 ± 1.3	0.0 ± 0.0	5.0 ± 5.3
	Total	147	7.8 ± 3.1	7.2 ± 3.1	52.2 ± 13.5	51.3 ± 15.9	60.8 ± 23.4	71.6 ± 33.3	74.8 ± 28.6

*WAB subscore are the mean ± SD. Maximal scores for each subscores are as follows: Content (10), Fluency (10), Y/N (60), Word Rec (60), Seq Command (80), Repetition (100)*.

a *WAB naming subtests were not administered in the Neurodegenerative group; hence AQ severity scores were not generated*.

b *The last clinical assessment for each patient is represented in the neurodegenerative cohort, given its progressive nature. The earliest clinical assessment is represented in the stroke and post-resective cohorts, given their nature of recovery*.

**Table 2:** Contributions of WAB subscores to each principal component dimension.

	**Dimensions**			
	**1**	**2**	**3**	**4**
Content	−0.18	0.09	0.10	0.05
Fluency	−0.15	0.31	0.11	−0.23
Y/N	−0.16	−0.22	0.31	0.06
Word Rec	−0.17	−0.16	−0.07	−0.09
Seq Commands	−0.17	−0.11	−0.27	−0.20
Repetition	−0.17	0.11	−0.15	0.38

As seen in Table 2, the first dimension comprised of all six tasks nearly equally. This correlational structure suggests that the combination of all the tasks explained more variability in the data than any independent contribution of an individual task. Dimension 1 strongly correlated with the AQ (*R*^2^ = 0.96), suggesting that it represents clinical severity, such that lower values indicate near normal phenotypes and higher values indicate more severe phenotypes. Given that the AQ was derived from an equal representation of each domain (fluency, comprehension, repetition, naming and information content) (26), it is not surprising that Dimension 1 would reflect the AQ. Dimension 2 is composed of both positive and negative contributors, thus separating tasks involved in fluency and production from those involved in comprehension and semantic meaning. Thus, while Dimension 1 captures disease severity, Dimension 2 characterizes the quality of the aphasia, separating fluency from comprehension. In this study, we focus on these two dimensions to allow for a simplified and intuitive visualization of the combined data.

**Figure 1:**
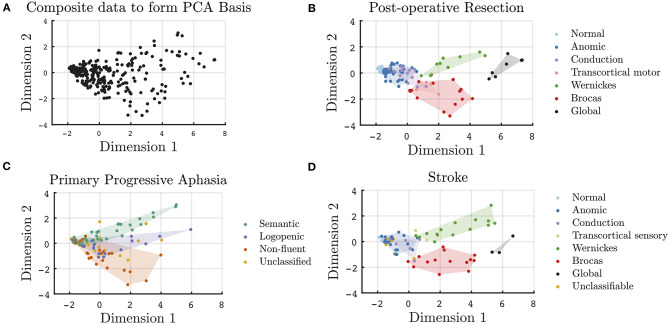
Cross-sectional visualization of WAB subscores onto a two-dimensional plane derived from a basis of 330 unique patients across three disease etiologies. **(A)** Projection of all data points used in the formulation of the common PCA bases onto Dimensions 1 and 2. WAB cross-sectional data for patient subgroups projected onto the common basis for all unique **(B)** post-operative, **(C)** PPA, and **(D)** stroke patients.

A two-dimensional plane that extends in the orthogonal dimensions of 1 and 2 captures 87% of the variability in the cross-population data. In Figure 1A, each patient used to create the common bases is projected onto the two-dimensional plane; each point represents a unique patient. Cross-sectional data of the post-resective, stroke, and PPA cohorts are plotted onto the same axes in Figures 1B–D. The post-operative (Figure 1B) and stroke (Figure 1D) patients are color coded by their WAB classification. Given that WAB subscores are used to formulate the two dimensions, as well as to classify aphasia subtypes for the lesional cohorts, the clustering of these clinical classifications within the two-dimensional space is an expected finding. However, the relative relationships across classical aphasias and primary progressive aphasia are clearly depicted in this visualization. Along dimension 1, this visualization follows clinical aphasia subtypes across severity with normal variants on one end of the spectrum and global aphasiacs on the other. Complementing severity scores, Dimension 2 is observed to add additional information on subtype. Patients with Wernicke's and Broca's aphasias are separated (*p* < 0.001) in Dimension 2 but not along Dimension 1 (*p* = 0.30). Those with transcortical motor aphasia are more closely clustered to those with Broca's aphasia, whereas transcortical sensory aphasic patients cluster more closely to those with Wernicke's aphasia, abiding by the separation of fluent and non-fluent aphasia types along Dimension 2. Of note, as the data is normalized prior to performing PCA, the relative values rather than absolute values provide insight on the relationship between phenotypes; the absolute values are nevertheless included in the figures to provide a method of comparison across plots.

In Figure 1C, the PPA cohort is color coded by their clinical classification. In this two-dimensional space, despite only utilizing subscores from a screening aphasia battery, the clustering of the clinically classified PPA variants was found to be preserved. Just as Wernicke's and Broca's aphasias are separated on Dimension 2, svPPA, lvPPA, and nfvPPA clusters are statistically different along Dimension 2. The lvPPA cluster is statistically different from svPPA (*p* < 0.001) and nfvPPA (*p* = 0.005) along Dimension 2, as well as svPPA from nfvPPA (*p* < 0.001). There was no distinction in the classification of PPA syndromes with respect to severity alone, i.e., along Dimension 1. When assessing only early PPA clinical presentations, as in Supplementary Figure 2, the separability between PPA variants is less significant. Early presentations of svPPA remain statistically separable from those of nfvPPA (*p* = 0.02) and lvPPA (*p* = 0.04) along Dimension 2; however, early presentations of nfvPPA are not statistically different from that of lvPPA (*p* = 0.71), given that additional specialized aphasia batteries are required for early PPA phenotyping. The weighted PCA technique did mildly improve the statistical separability of the early presentations of PPA, as shown in Supplementary Figure 3. In addition, as seen in Supplementary Figures 2, 3, the visualization of unclassified patients falling outside of the established clinical clusters is more apparent in later clinical presentations, as compared to early presentations.

### Longitudinal Study: Temporal Evolution of Aphasia During Disease Progression and Recovery

**Figure 2:**
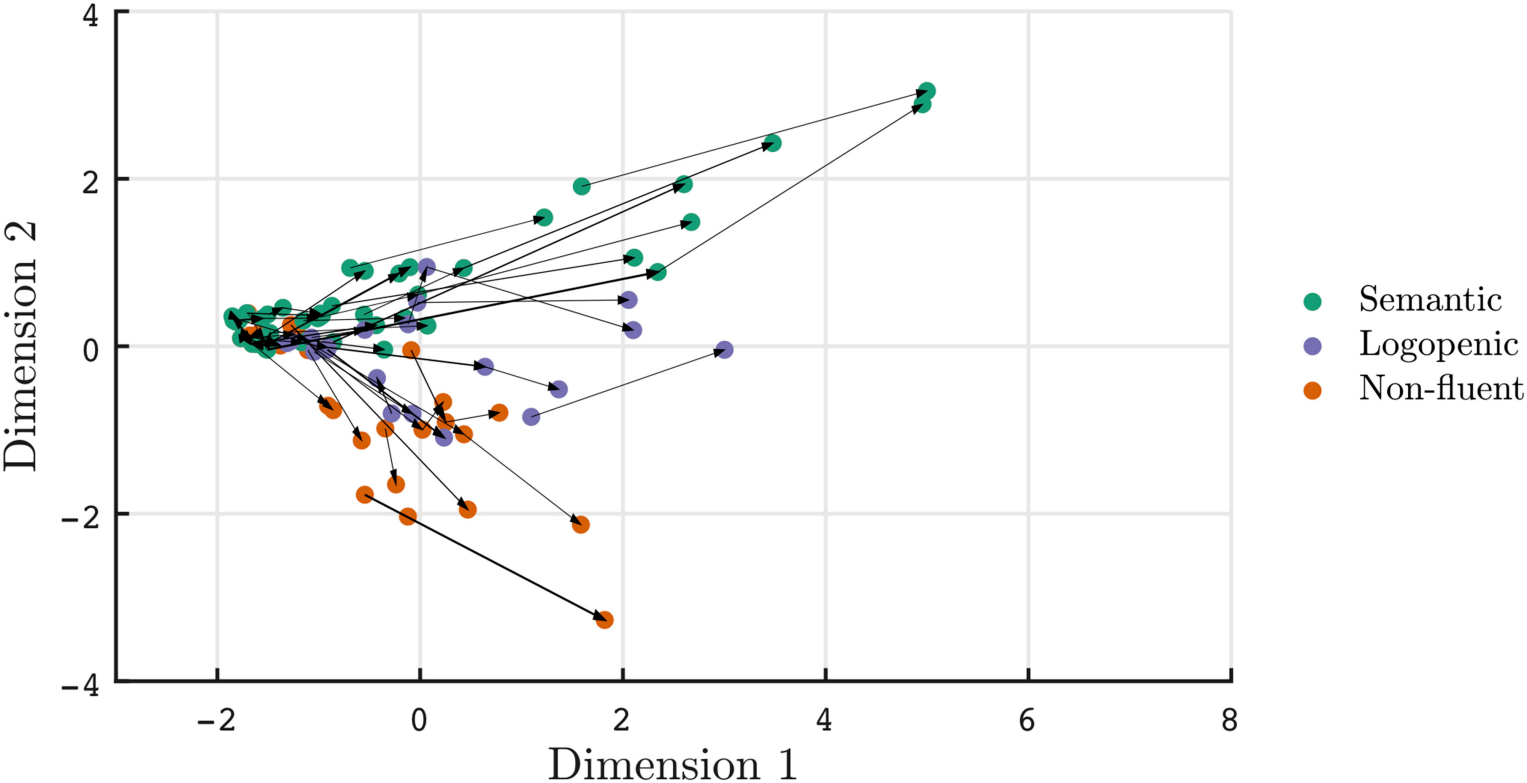
Visualization of PPA patient trajectories with each arrow connecting individual patients at each clinic visit. Patients meeting clinical classifications are depicted. Over time, aphasias phenotypes become more severe (i.e., increased values along dimension 1) and separable in quality (i.e., larger magnitudes along dimension 2).

**Figure 3:**
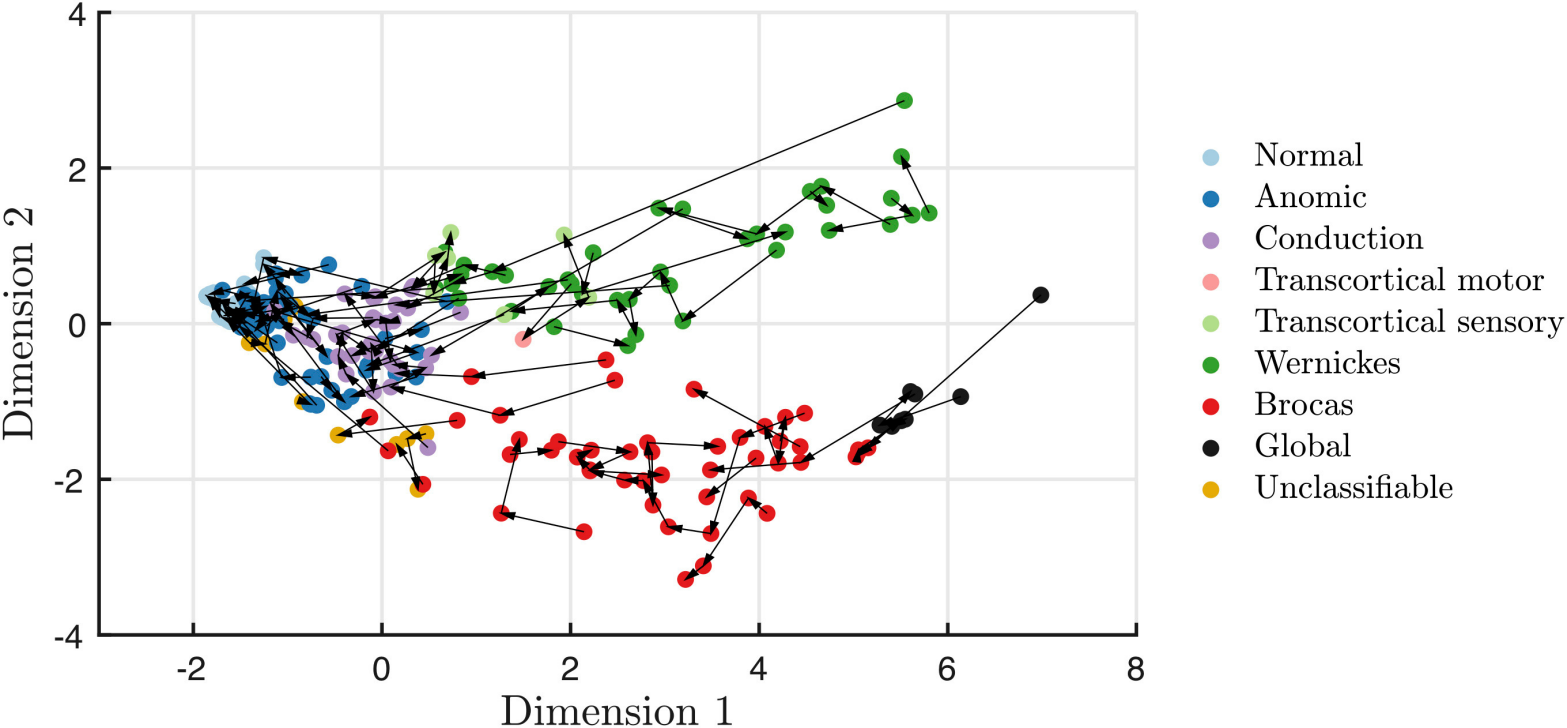
Visualization of stroke patient trajectories with each arrow connecting individual patients at each clinic visit. Over time, stroke recovery leads to more mild forms of aphasia, while still maintaining the fluency distinction within Dimension 2.

**Figure 4:**
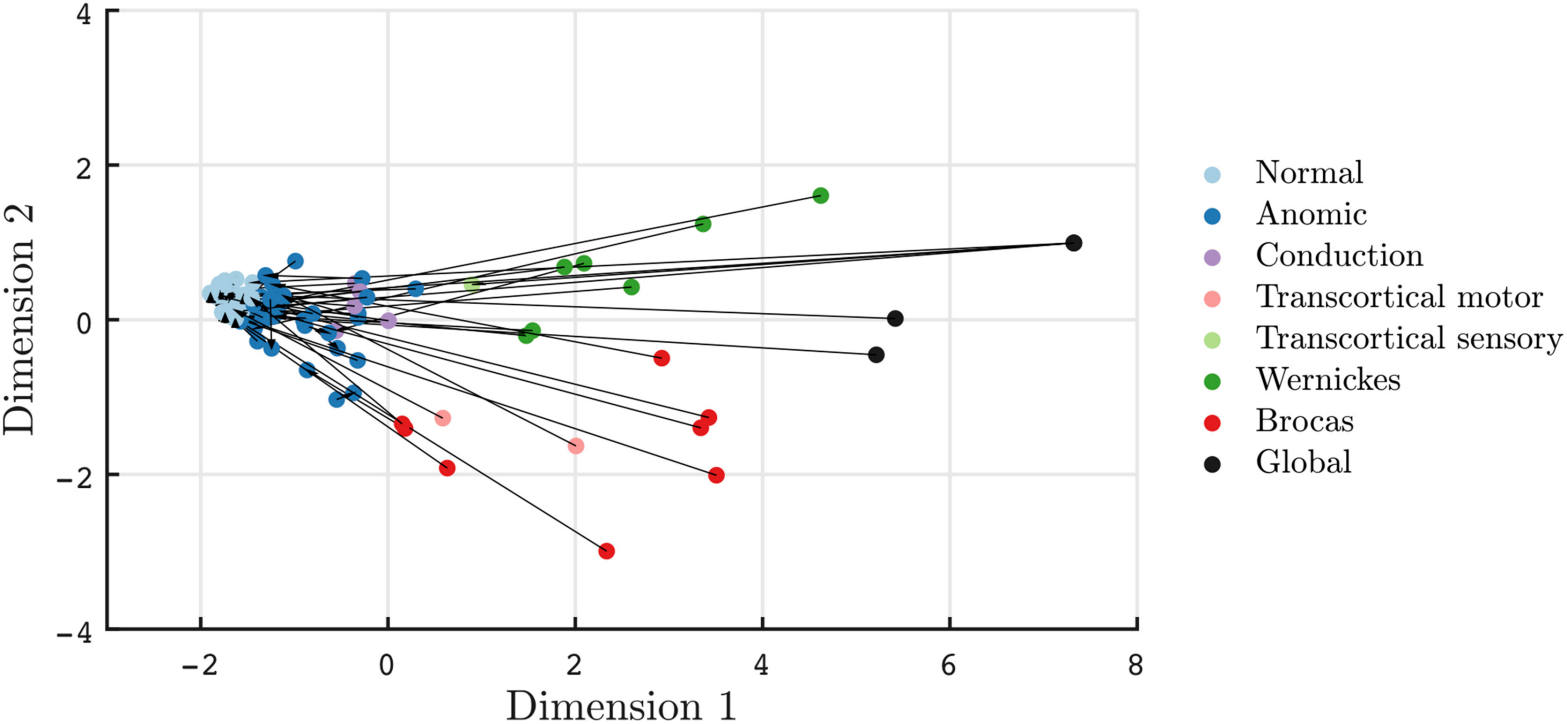
Visualization of transient aphasias in post-neurological surgery patients from 2 to 3 days to 1 month post-operative. Patients rapidly improve to normal and anomic phenotypes within 1 month of post-resective surgery.

Beyond cross-sectional data, the two-dimensional representation of aphasias can be useful in tracking and summarizing symptom evolution over time. In Figures 2–4, a subset of patients with longitudinal data are projected onto the common basis described in Figure 1. Arrows are used to connect unique patient encounters for individual patients over time. In Figure 2, the progression of PPA across clinic visits is demonstrated by increasing severity across Dimension 1, as well as higher absolute values for nfvPPA and svPPA variants along Dimension 2. Furthermore, individual tracings suggest that some lvPPA have tendencies toward nfvPPA or svPPA, particularly in the later course of the disease. The mean time between clinic visits for the PPA cohort is 15.6 months (SD = 6.6, median = 13 months). A cross sectional depiction of early vs. late clinical presentation of PPA patients and the spread of aphasia phenotypes is visualized in Supplementary Figure 2. Scores along dimensions 1 and 2 and vectors of change are demonstrated to provide summary metrics to supplement original subscores.

Recovery in aphasia syndromes for stroke and post-resective patients is seen in Figures 3, 4, respectively. The quantitative scores and relative locations within the two-dimensional space, depicting the subtype of aphasia, can be tracked through the recovery period. For instance, in this sampled patient population, global aphasics are found to recover toward a trajectory similar to that of Broca's aphasics as compared to that of Wernicke's aphasics. Over time, patients on average converge toward less severe aphasias. In addition, recovery tends to abide by the natural division of Dimension 2, maintaining separation of fluent and non-fluent aphasia types, as observed in prior studies (2, 27). The mean time between clinic visits for the stroke cohort is 12.7 months (SD = 16.5, median = 5.0 months). Rapid recovery is visualized in the post-resective patient cohort in Figure 4, in which arrows are connecting individual patients from 2 to 3 days to 1-month post-operative; the cross-sectional visualization of the recovery process is depicted in Supplementary Figure 4. From this diagram, nearly all patients who suffered a transient aphasia, even those of high severity, improved to within normal limits or to anomic aphasia.

## Discussion

By evaluating a large cohort of patients suffering from stroke, neurodegenerative, and post-resective aphasia, we have provided a *two-dimensional* framework that can visualize language impairment across a variety of disease etiologies. While the language tool assessment shared across three cohorts highlights the similarities of aphasia phenotypes across disease etiologies, as with prior dimensionality reduction studies (22), the proposed framework using only two dimensions also provides sufficient granularity to preserve clinical classifications across all cohorts. This visual method of characterizing an aphasia phenotype provides a simplified and intuitive method of rapidly assessing aphasia phenotypes across populations and time.

### Interpretation of the Two-Dimension Model Across Disease Etiologies

The two-dimensional spectrum provides a method to identify behavioral similarities across patients from different disease etiologies. The scores along dimension 1 and 2 are shown to be descriptors of an aphasia, characterizing both the severity and the quality of aphasia. The scores and relative locations of established clinical phenotypes are shaded in Figures 1B–D. While Dimension 1 strongly correlates with severity, Dimension 2 separates Broca's from Wernicke's aphasia, as well as the three variants of PPA. Considering the contributions of each WAB subtype as in Table 2 and the WAB scoring system, in which lower WAB subscores signify worse performance, aphasic patients with tendencies toward poor production would have stronger net negative magnitude in Dimension 2, whereas patients with tendencies toward intact fluency but poor comprehension would have stronger positive magnitudes in Dimension 2. Magnitudes near zero may imply minimal symptoms, as in the within-normal-limits or anomic patients, or it may indicate combined positive and negative contributions, such as in the global aphasics. While the interpretation of latent structures derived from PCA has limitations, the clustering of clinical phenotypes can help provide intuition to each dimension. In particular, the distinction in the deficits reflected in Dimension 2 has historically aligned with frontal and temporal pathology or dorsal and ventral streams (28). Dimension 1, representing general aphasia severity, is derived from all subscores and may have broader considerations of functional and structural networks, in addition to their localization (29).

In comparing across disease etiologies, fluent and non-fluent forms of aphasia for both lesional and neurodegenerative etiologies are shown to similarly separate along Dimension 2. Both Broca's aphasics and severe nfvPPAs localize to similar areas within the two-dimensional space, suggesting more similarity with each other than with other phenotypes. nfvPPA is characterized by effortful speech, phonetic or motor speech impairment compatible with apraxia of speech, dysarthria and/or by agrammatism. Broca's aphasia in the context of stroke aphasia is also characterized by halting, effortful speech with agrammatism and apraxia of speech. The behavioral similarity mirrors the neuroanatomical similarity, in which peak atrophy for patients with nfvPPA localizes to the left posterior inferior frontal gyrus and adjacent areas (3). Investigation at higher dimensions or with more detailed behavioral assessments would enable discernment of behavioral differences (22). For instance, studies of nfvPPA have shown that grammar and fluency can be dissociated; case examples of patients with nfvPPA were found to have near normal fluency but impaired grammar and vice versa. In the current study, the dissociation between grammar and fluency was not seen in any of the dimensions, given that datasets obtained across all disease etiologies did not include grammar or naming specific assessments. Higher dimensions may also provide insight in other sample-based sources of variance, such as behavioral variation related to multilingualism, which would be of interest for future study. However, here, the two-dimensional framework proposed here is intended as a simplified tool for the rapid assessment of an aphasia phenotype generalizable to etiology at a single time and across time. Constructed on screening aphasia testing, the framework focuses on the similarities across etiology, and yet enables the granularity required for clinical separability of classical phenotypes within etiology.

Similar behavioral associations can be drawn in other regions of the two-dimensional model. For instance, lvPPAs are seen to resemble conduction and anomic aphasias. While the extent to which lvPPA is a distinct entity has previously been a subject of debate (30), here it clearly emerges as a separate group within Dimension 2.

Within the two-dimensional model, svPPAs were found to overlap with Wernicke's and transcortical sensory aphasias, essentially ventral to the Sylvian fissure, suggesting that their behavioral phenotypes are overall more similar to one another than with other phenotypes. Comparison studies between the svPPA and Wernicke groups have indicated qualitatively different semantic deficits, including relative sparing of sentence comprehension or sensitivity to frequency/familiarity in semantic dementia, as compared to Wernicke's aphasia (6, 31, 32). While such qualitative differences are again not the focus of this study, the method here may provide an effective screening tool for more detailed assessments across disease etiologies. For instance, patients across etiologies with similar scores along Dimension 1 and 2, as derived from a small subset of WAB data, would be candidates for more detailed linguistic and anatomical comparisons given aphasias of similar characteristics and severities.

Many studies have illustrated stroke aphasias via a multi-dimensional template (33–35), displaying cross-sectional data on an axis of phonology vs. semantic factors. Other studies have attempted to subtype PPA variants on a two dimensional template comprised of grammaticality and word comprehension, based on the clinical identification of orthogonal tasks for subtyping (36, 37). Only one prior study, Ingram et al. (22), proposed a multidimensional cross-cohort platform, composed of four dimensions. As compared to Ingram et al. (22), this study focused on the development of a two dimensional platform using a screening aphasia battery to provide a *single* framework for characterizing phenotypes. Because this study employed two native dimensions rather than a four factor rotated solution (22), the interpretations of the dimensions were distinct. The four-axis model (22) features phonology, semantics, motor production, and visuoexecutive functioning on its axes, whereas the simplified, two-axis model proposed here features aphasia severity and quality, e.g., separating fluency from comprehension in opposing directions. Here, we demonstrate that even using a reduced number of subscores and a two-dimensional platform, classical disease phenotypes were statistically distinguishable and could be tracked over time.

### Implications: Visualization of the Spectrum of Aphasias

Defining aphasia subtypes is essential for understanding patient symptomology and associating symptoms with disease pathophysiology. However, a challenge of developing clinical classification is to account for diverse phenotypic variability. Within the stroke literature, the WAB and the BDAE have been used for many decades. Despite even these two standardized classifications, there is often disagreement in the classification of patients with aphasia (38). From the PPA perspective, current classification schemes are based on the assessment of over 10 different components of language (8). Despite widespread acceptance of these classifications, several studies noted that up to 40% of PPAs are not classifiable by the current guidelines, the majority meeting the criteria of multiple PPA variants (11, 36, 39–41). As with the four-dimensional model (22), the heterogeneity of behavioral characteristics that challenge current classification schemes in multiple disease etiologies can be elucidated in the two-dimensional model, based on the continuum of the two-dimensional space.

As seen in Figure 1, the spectrum of phenotypes within a single clinical classification is apparent for both lesional and neurodegenerative etiologies. In Figure 1C, three PPA variants are separable in Dimension 2; however, each variant has considerable heterogeneity, as seen by the spread in severity and quality scores, even when evaluating with a screening language assessment. When considering unclassifiable PPA patients, many fall within the bounds of the early presentations of PPA; however, several are seen to fall in between the clinical clusters along Dimension 2, as shown in yellow in Figure 1C. These depictions highlight that, in both stroke and neurodegenerative disorders, clinical classifications are broad syndromic descriptions that include patients who can have different relative impairment of motor speech and/or grammatical linguistic difficulties. For instance, syndromes that isolate motor speech or grammatical impairments depend on localization and extent of the lesion, as well as type of tissue involved, i.e., white vs. gray matter (42–44).

Another challenge to current classification schemes is that diagnoses are based on one time point rather than accounting for evolution in time. In the PPA literature, this limitation has been most commonly discussed in regards to lvPPA (39). For instance, patients in their prodromic state of nfvPPA or svPPA may be misclassified as lvPPA (39, 45). In Figure 2, several trajectories display tendencies toward misclassification, as some patients classified as lvPPA are seen to have trajectories toward nfvPPA or svPPA over time. As the epicenter of disease in lvPPA can vary between the angular gyrus or superior posterior temporal gyrus (46), the trajectories of lvPPA may reflect progression within frontal and temporal language networks.

It is important to note that while this model may provide broad insight into syndrome classifications and trajectories with respect to neighboring clinical clusters, the trajectories are determined from a subset of behavioral scores. Further investigation with expanded language batteries applied across cohorts and associated imaging studies are required to probe underlying neurobiology and cortical connectivity.

### Implications: Quantitatively Tracking Disease Progression Over Time

Studies have utilized data-driven techniques, such as PCA (22, 34, 47, 48), ANOVA (49) and hierarchal cluster techniques (41), for the characterization of aphasias. The application of quantitative methods for assessing behavioral phenotypes across different aphasia etiologies using a two-dimension platform and over time are novel contributions. By employing WAB subscores as a screening tool, summary scores of a patient's severity and aphasia subtype can be plotted along a landscape of clinical aphasia syndromes. Furthermore, averaged trajectories can be ascertained through computation of unit vectors of aphasia recovery or progression, as in Supplementary Figure 5.

A simplified framework of visualizing aphasias has broad clinical applications including tracking quantitative metrics of language over clinic visits to enable clearer communication to families and other providers (50) or visualizing two-dimensional outcome measures and trajectories with respect to risk factors (18) or therapies (51). Furthermore, a cross-etiology platform can be used as a screening method to identify patients from multiple disease cohorts with greatest resemblance, as an initial step prior to pursuing more detailed testing and subsequent neurobiological comparisons. Using data-driven approaches, this study presents a *simplified, two-dimensional model* visualization that can provide rapid intuition on the severity and general quality of the aphasia over time and is generalizable to multiple patient populations.

## Data Availability Statement

The datasets presented in this article are not readily available because there are ongoing research efforts that are using these data. Requests will be considered on a case by case basis. Requests to access the datasets should be directed to Joline M. Fan, joline.fan@ucsf.edu.

## Ethics Statement

The studies involving human participants were reviewed and approved by UCSF IRB Committee. The stroke cohort data were provided via a Cooperative Research and Development Agreement between the VANCHCS and UCSF. The patients/participants provided their written informed consent to participate in his study.

## Author Contributions

JF performed the analysis and drafting of the manuscript. MG-T, ND, and EC assisted with the data acquisition, analysis, editing, and review of the manuscript. BM and MB assisted with the overall interpretation of the data and the review of the manuscript. All authors contributed to the article and approved the submitted version.

## Conflict of Interest

The authors declare that the research was conducted in the absence of any commercial or financial relationships that could be construed as a potential conflict of interest.
