# Supplementary material for: Data-Driven, Visual Framework for the Characterization of Aphasias Across Stroke, Post-resective, and Neurodegenerative Disorders Over Time
Source: Front Neurol. 2020 Dec 29;11:616764. doi: 10.3389/fneur.2020.616764 (PMC7801263; doi:10.3389/fneur.2020.616764)
Supplement: Supplementary file 1 [file Data_Sheet_1.pdf]

Supplementary Material

Weighted PCA applications:

In order to account for the effect of sampling population on the determination of principal components (PCs), a weighted PCA[18,19] was also performed to allow for equal representation of clinically distinct aphasia types, regardless of disease etiologies. Unclassified patients were not included in the development of the common bases. Data across all disease cohorts were combined to compute a common weighted mean and standard deviation, by which all patient data was normalized. The PCs were then obtained via computing the eigenvectors of a weighted covariance matrix, i.e. $\frac{1}{\sum w_i}\mathbf{X}^{\mathrm{T}}\mathbf{W}\mathbf{X}$, where **X** is the normalized data matrix with columns representing WAB features and rows representing observations, and **W** is the diagonal matrix of observational weights, w_i. As with the original PCA calculation, each patient was represented once, as determined by the expected evolution of the cohort. Findings and relative landscapes of the data were found to be consistent with the non-Weighted PCA methods.

1 Supplementary Figures

	Dimensions			
	**1**	**2**	**3**	**4**
**Content**	-0.18	0.12	0.02	0.03
**Fluency**	-0.15	0.32	-0.12	0.17
**Y/N**	-0.16	-0.23	0.17	0.32
**Word Rec**	-0.17	-0.12	-0.15	-0.10
**Seq Commands**	-0.17	-0.14	-0.23	-0.13
**Repetition**	-0.17	0.07	0.31	-0.25

Supplementary Table 1: The contributions of WAB subscores to each principle component dimension is displayed above. The variability explained by each dimension is as follows: Dimension 1 (74.4%), dimension 2 (10.8%), dimension 3 (5.4%), dimension 4 (3.0%).

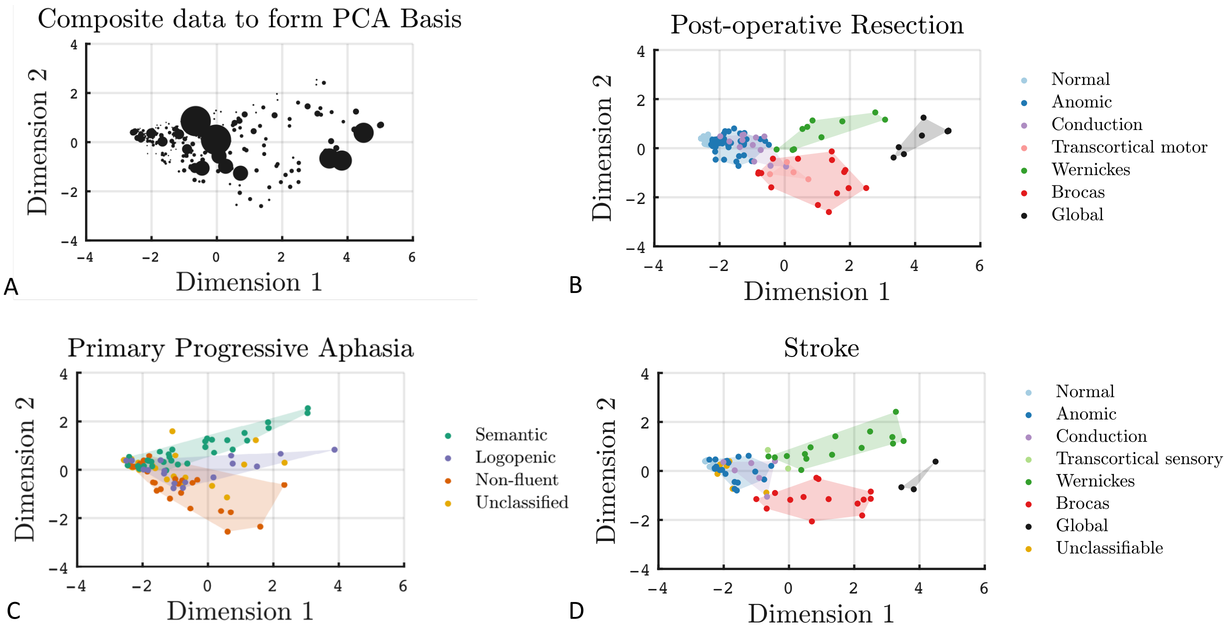

Supplementary Figure 1: A) Demonstration of the projection of individual data points used in the formulation of the common PCA bases, constructed from a weighted PCA. The relative weighting of each clinical aphasia subtype is scaled to the size of the data point. WAB cross-sectional data for patient subgroups were projected onto the common basis for B) post-operative, C) PPA, and D) stroke patients. The relative positions within the two dimensional space are consistent with the non-weighted PCA formulation, described in the paper.

Using non-weighted PCA

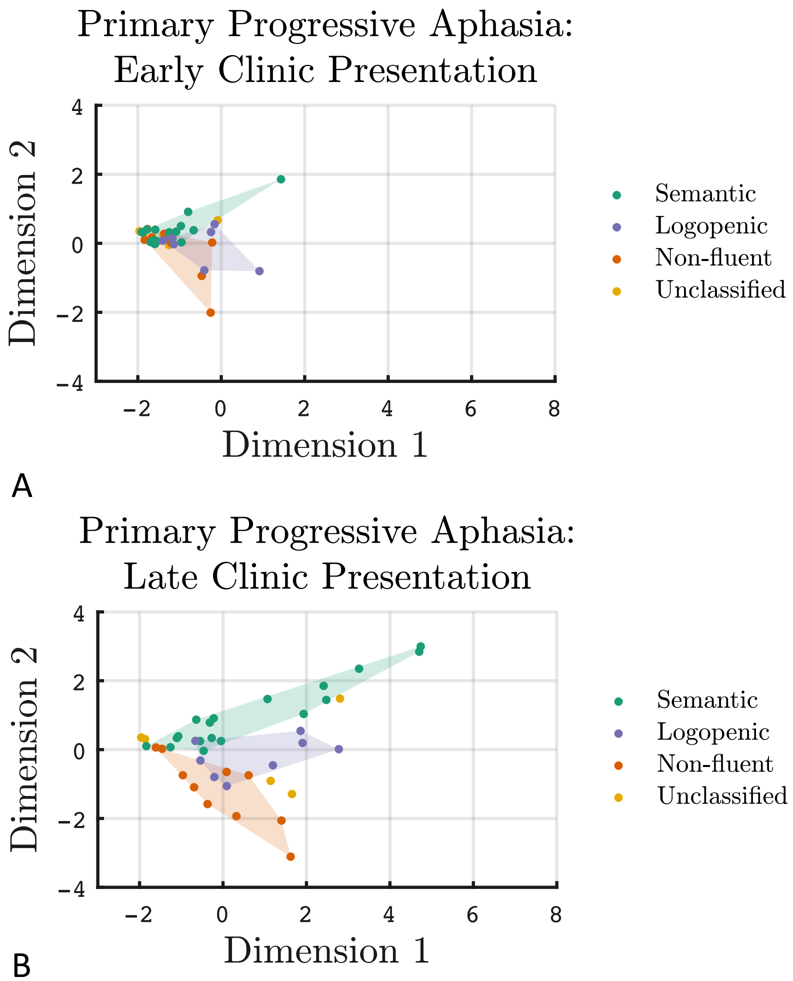

A

B

Supplementary Figure 2: Visualization of the spread of WAB subscores for patients with PPA using **non-weighted PCA** during (a) early vs (b) late clinic presentations, as an extension to Figure 2. Only PPA patients with time-series data are included in this diagram, including those who are unclassified. In comparison to early presentation, there is increased separability with the WAB screening assessment along dimension 2 at later clinic presentations. Along dimension 2, svPPA is statistically separable from that of nfvPPA ($p = 0.02$) and lvPPA ($p = 0.04$); however, early presentations of nfvPPA are not statistically different from lvPPA ($p = 0.71$).

Using weighted PCA

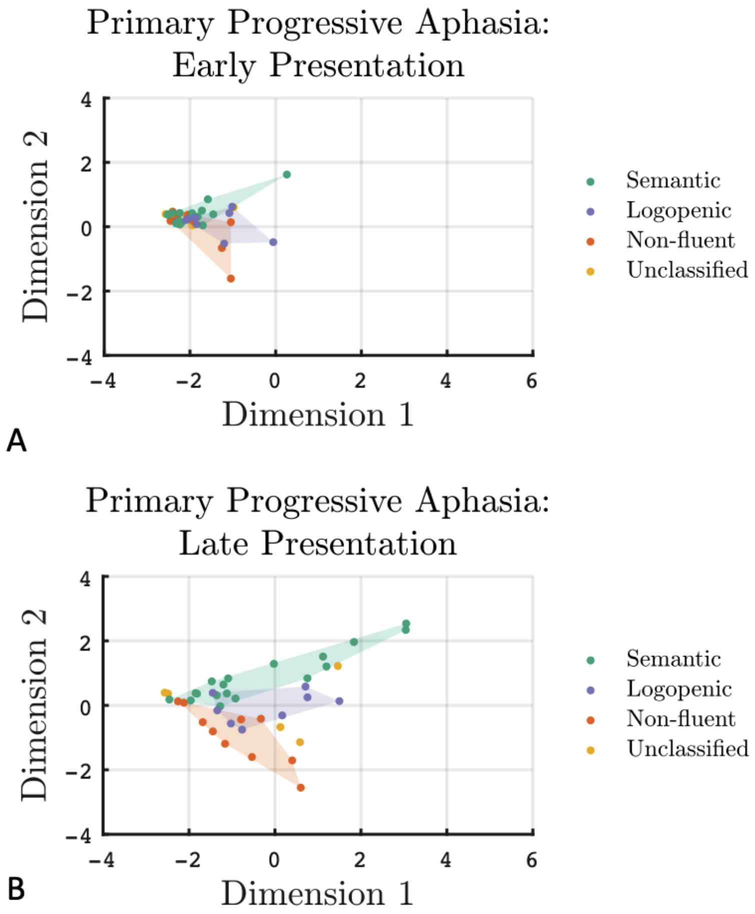

Supplementary Figure 3: Visualization of the spread of WAB subscores **using weighted PCA** for patients with PPA during (a) early vs (b) late clinic presentations, as a supplement to the non-weighted PCA evaluation. Again, only PPA patients with time-series data are included in this diagram, including those who are unclassified. As compared to non-weighted PCA, early presentations of PPA variants were overall more statistically separable. Early presentations of svPPA remained statistically separable from that of nfvPPA ($p < 0.001$) and lvPPA ($p = 0.011$) along dimension 2; however, early presentations of nfvPPA remain not statistically different from lvPPA ($p = 0.124$) via this clustering technique.

Using non-weighted PCA

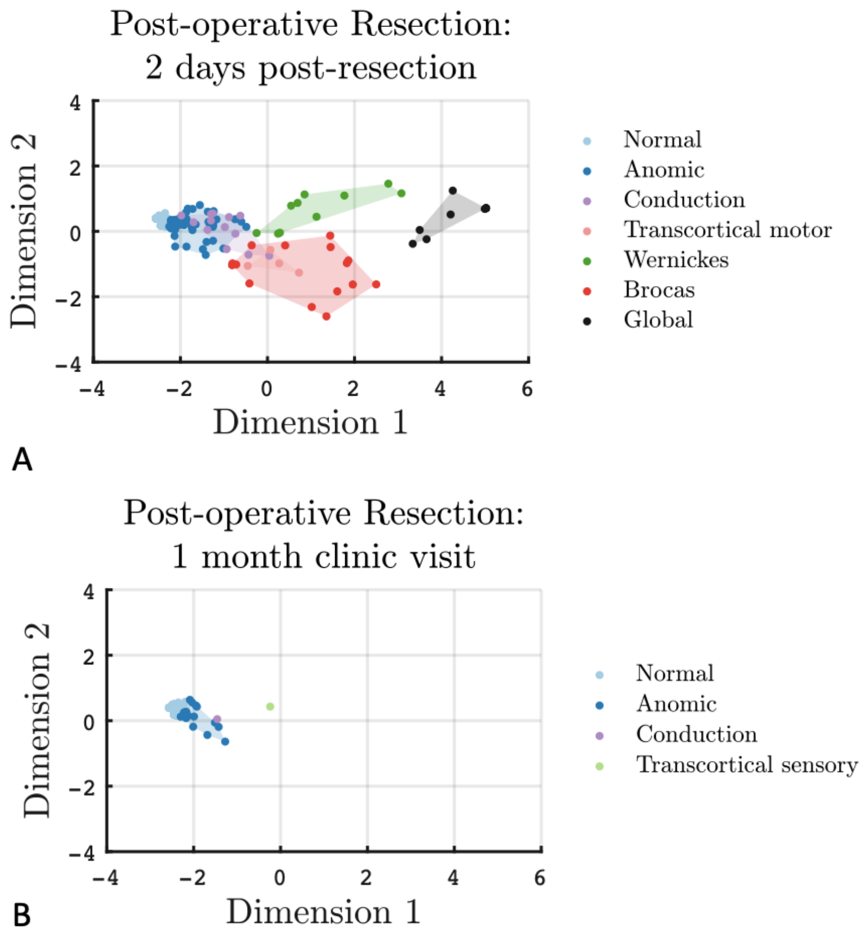

Supplementary Figure 4: Visualization of the spread of WAB subscores for post-operative patients from (a) 2 days post-resection to (b) 1 month post-resection, as an extension to Figure 4. Within one month, there is near normalization of language dysfunction.

Weighted unit vectors of aphasia progression or recovery

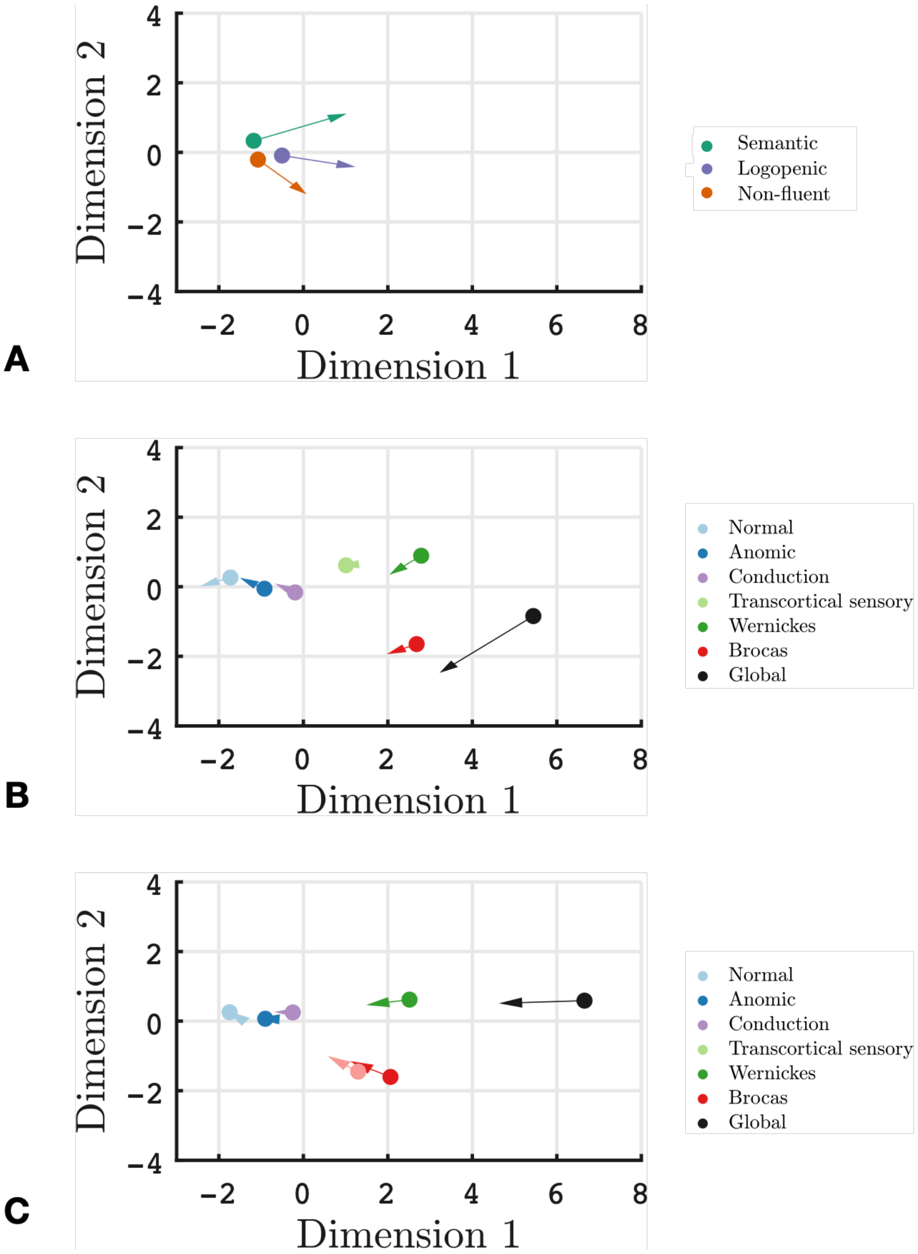

Supplementary Figure 5: Visualization of unit weighted vectors of change for a) PPA cohort over 24 months, b) stroke cohort over 24 months and c) post-resective cohort over a week. Unit vectors are determined by a weighted average of all vectors within a class; weights are determined by the duration between clinic intervals, based on the premise that longer durations give rise to more reliable trajectories. Of note, because expected trajectories are non-linear over time (e.g. aphasia recovery in the stroke cohort may first manifest faster recovery followed by slower recovery) and trajectories are averaged over all available time courses, the unit vector may have a tendency to underestimate recovery in the stroke cohort (as compared to earlier time courses of recovery) or potentially overestimate PPA progression (as compared to later time courses). These graphs provide a proof of concept that a two-dimensional framework, as such, may provide insight into expected disease progression depending the current phenotype.
